# Interaction between a robot and Bunimovich stadium billiards

**DOI:** 10.1038/s41598-022-08897-4

**Published:** 2022-03-23

**Authors:** J. V. A. Vasconcelos, L. H. Miranda-Filho, A. J. F. de Souza, A. R. de C. Romaguera, A. L. R. Barbosa

**Affiliations:** Departamento de Física, Universidade Federal Rural de Pernambuco, Rua Manoel de Medeiros, s/n - Dois Irmãos, 52171-900 Recife, Brazil

**Keywords:** Statistical physics, Engineering, Applied physics

## Abstract

The robot–environment–task triad provides many opportunities to revisit physical problems with fresh eyes. Hence, we develop a simple experiment to observe chaos in classical billiards with a macroscopic 3.38-m long setup. Using a digital video camera, one records the dynamic time evolution of the interaction between a robot and Bunimovich stadium billiards with specular reflection. From the experimental time series, we calculate the Lyapunov exponent $\lambda$ as a function of a geometric parameter. The results are in concordance with theoretical predictions. In addition, we determine the Poincaré surface of section from the experimental data and check its sensitivity to the initial conditions as a function of time.

## Introduction

Chaos is a theory that has ramifications in all of science^1–3^. Lorenz’s work in meteorology^4^ is the modern foundation stone of chaos theory. The influence of chaos theory extends beyond physics and mathematics to include biology, chemistry, communications, engineering, cryptography, and robotics^5–14^. It provides a collection of concepts and methods to analyze a novel behavior emerging in a wide range of disciplines^1^. The hallmark of chaos is the super-sensitivity of a nonlinear system to its initial conditions, which makes classical dynamics appear unpredictable over long periods of time^3^.

**Figure 1:**
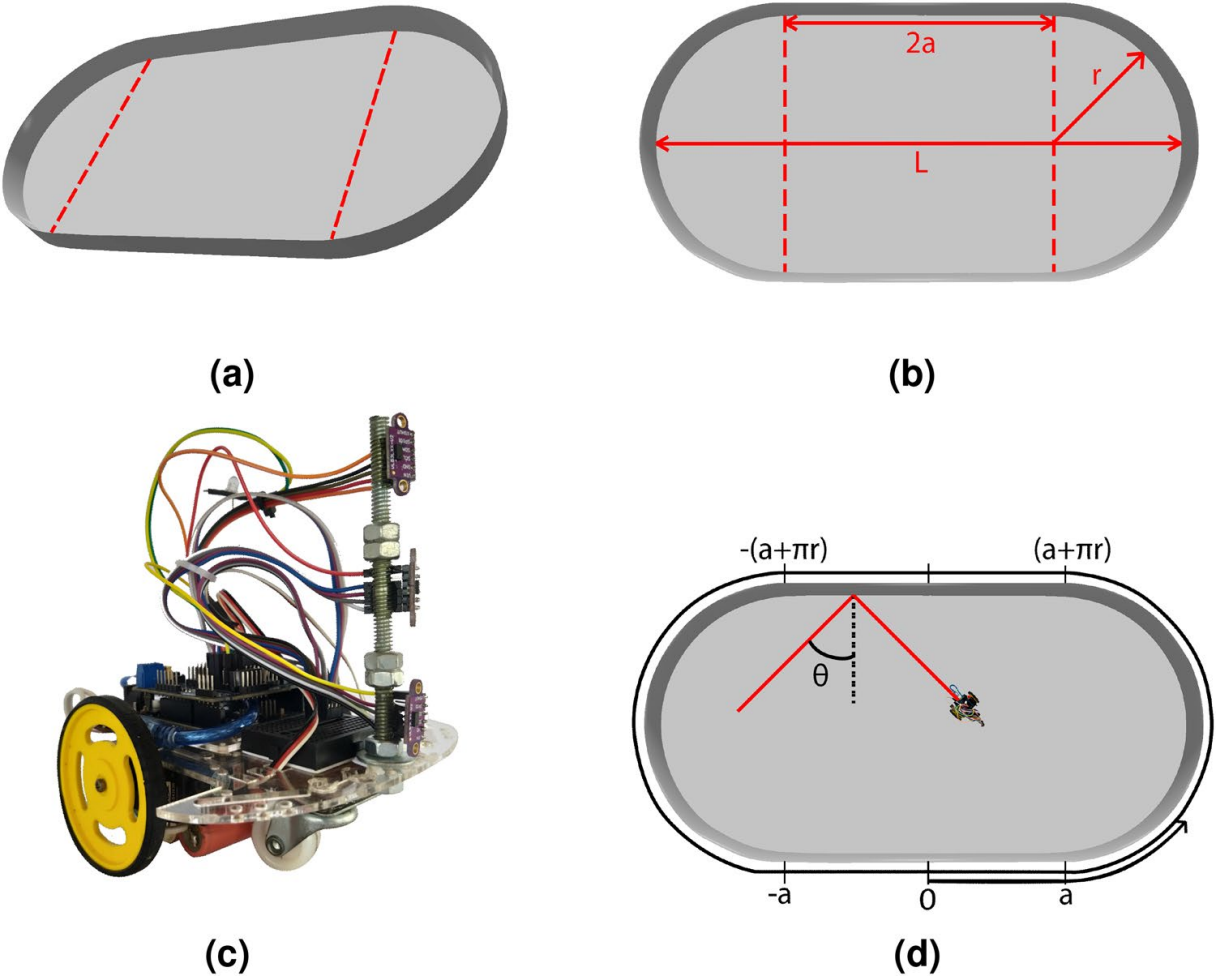
(**a**) Panoramic view of the stadium. The walls are 25 cm high and the fixed area in the experiment is *A* = 2.57 $\hbox {m}^2$. (**b**) Geometrical parameters of the Bunimovich stadium billiards. The red dashed vertical lines separate the semicircles of radius *r* from the central rectangle with side length 2*a*. The total length is $L=2(r+a)$. (**c**) Arduino robot used in the experiment. The dimensions of the robot (*l*, *w*, *h*) are 14 cm, 12.5 cm, and 16 cm, respectively. (**d**) The stadium in Birkhoff coordinates (*p*, *s*), where $p\in [-2a-\pi r,+2a+\pi r]$ lies on the boundary, and the projection velocity parameter $s= \sin \theta _{i}\in [-1,+1]$, where $\theta _i$ is the incident angle.

The dynamics of billiards is probably the simplest system in which chaos emerges^10,15^. This system has two axes of symmetry which can be also used to increase the number of trajectories in it. The motion in billiards consists of a sequence of straight flights interrupted by specular reflections. For example, in circular stadium billiards^16^ (CSB), the particle’s evolution is insensitive to the initial conditions. In this case, the sequence of straight flights and reflection angles produce a regular time series. Consequently, the particle carries out periodic orbits, visiting only a part of the internal area of the stadium. However, when the boundary is shaped as in Bunimovich stadium billiards^17^ (BSB), as illustrated by Fig. 1a, or in Sinai stadium billiards^18^ (SSB), the sequence of straight flights and angles is a non-trivial time series. This means, then, the dynamics is sensitive to the initial conditions, is non-periodic, and thereby the particle accesses all the regions of the stadium. The BSB and SSB are examples, therefore, of ergodic dynamics^19^, which assert that, under certain conditions, the time average of a function along the trajectories exists almost everywhere and is related to the space average. In addition to the ergodic properties, BSB is also a mixing system with Kolmogorov property^19^ and Bernoulli property^20^, which means that regular and chaotic dynamics coexist.

The Lyapunov exponent (LE), historically denoted by $\lambda$, is an objective measurement of chaos. This quantity is related to the linear stability of a trajectory. When $\lambda > 0$, there exists at least one direction in phase space in which the dynamics is unstable implying chaotic behavior. The standard method for calculating $\lambda$ is the so-called tangent space method that consists in the integration of the system’s equation of motion with its linearized versions considered in successive time steps^21^. From this, and a generic initial perturbation, we appropriately estimate the most expanding direction of deviations.

In certain situations, the linearized version of the state equations is unavailable due to presence of discontinuities. In such cases, cloned dynamics method^22^ can be employed. Also, when one has only a time series of observations, the algorithm proposed by Wolf et al.^23^ is an alternative; their method is based on phase space reconstruction techniques using delay coordinates. There is a loss of precision, unfortunately, when using the clone’s and Wolf methods, since one obtains the Jacobian by approximated reconstructions.

By using tangent space method, Benettin and Strelcyn^9^ developed numerical calculations to obtain the LE for a perfect BSB boundary defined by the half side length *a* and the circular radius *r*, as illustrated by Fig. 1b. For a perfect BSB with constant area, $A=\pi r^2+4ra$, the LE is null when $a=0$ and increases quickly with $\gamma =a/r$ reaching a maximum value of $\lambda \approx 0.43$ at $\gamma = 1$. Afterward, it decays slowly as $\gamma \rightarrow \infty$. Note that, when $\gamma = 0$ the billiards is a perfect CSB while for $\gamma \rightarrow \infty$ the perfect BSB behaviors as a perfect rectangular bordering, both cases being non-chaotic. These results have been numerically verified^10,24^, but we are not aware of any direct experimental realization of such an idealized system.

We can mimic the classical dynamics of particles in billiards using, for example, microwave cavities^25^, optical systems^5^ and robotics^14,26,27^. Optical systems are a common research topic in fundamental sciences, whereas robot–environment experiments are mainly treated in applied sciences and engineering^28–30^. Here, we aim to show that the robot–environment interaction might be a suitable platform to study the fundamental science of classical dynamics.

In this work, we experimentally mimic the classical dynamics of a single particle in standard CSB and BSB boundaries as a robot–environment interaction. Arduino robot used in the experiment is showed in the Fig. 1c. Our experimental setup can provide all the measurements required to write the Jacobian for the perfect BSB. As a result, we obtain the LE using the tangent method for six different values of $\gamma$ for BSB keeping its area fixed in a 3.38 meters long setup, see Table 1. We confront our experimental results with forecasts of Benettin and Strelcyn^9^, which are proven to be completely compatible values. As a control on our experimental setup, we determine the Poincaré surface of section (SOS)^31–33^ for standard CSB and BSB using Birkhoff coordinates^34^, as illustrated by Fig. 1d. The SOS characterizes all system features in phase space. The geometrical symmetries of the stadium billiards and of the particle’s dynamics are intrinsically connected and they manifest as symmetries in the Poincaré SOS. The reflection symmetry of the BSB, the horizontal and vertical axes, makes left-right symmetric patterns in Poincaré SOS while the time reversal symmetry^35^ of the dynamics makes top-bottom symmetric patterns in Ponicaré SOS. Even irregular boundaries, with roughness, leads in top-bottom symmetric patterns thanks to time reversal symmetry. Those symmetries are presented in Fig. 3 as will be discussed later. Despite the geometrical and time-reversal symmetries, closed trajectories behave in a different way. They produce single points in Poincaré SOS usually surrounded by blank regions. In addition, to fully describe the dynamics of the billiards, we also present the time evolution of the paths of the robot in phase space in the Poincaré SOS for the robot’s trajectories.

## Results and discussions

**Figure 2:**
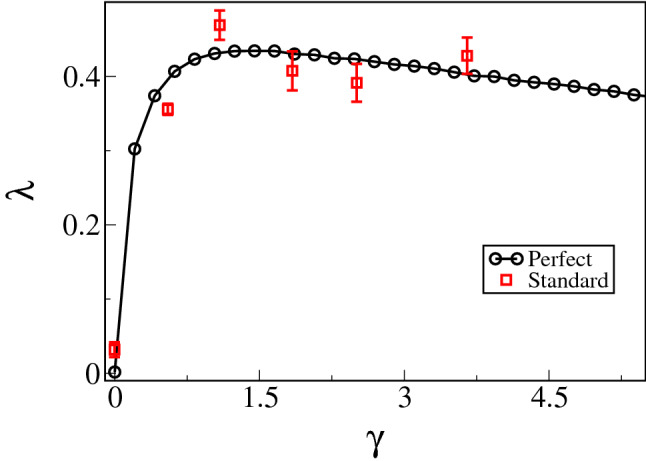
Lyapunov exponent ($\lambda$) as a function of the control parameter $\gamma =a/r$. The black open circles are the numerical results for the $\lambda$ for perfect BSB. The red open squares are the experimental results for $\lambda$ in a standard BSB.

Figure 2 shows the behavior of the LE as a function of the control parameter ($\lambda ~vs.~\gamma$). The black open circles are numerical results for a perfect BSB, which is illustrated by Fig. 1b. Here we numerically estimate the LE by following the dynamical evolution in the tangent space^22^, through an event-driven integration scheme. We have also observed that $5 \times 10^4$ collisions with the stadium’s boundary turned out to be enough for the convergence of the LE. Each numerical data point corresponds to an average of one hundred different initial conditions. Our numerical results agree with those first reported by Benettin and Strelcyn, see Fig. 4 of Ref.^9^.

**Table 1:** Geometrical parameters used for perfect and standard BSBs, Fig. 1b.

*r* (cm)	*a* (cm)	*L* (m)	$\gamma$	$\gamma _{eff}$	$\lambda _{p}$	$\lambda _{s}$
90.0	0.0	1.80	0.00	0.00	$0.00 \pm 0.01$	$0.03 \pm 0.03$
70.0	37.0	2.14	0.53	0.55	$0.40 \pm 0.01$	$0.36 \pm 0.01$
60.0	60.0	2.40	1.00	1.08	$0.43 \pm 0.01$	$0.47 \pm 0.02$
50.0	89.0	2.78	1.79	1.84	$0.43 \pm 0.01$	$0.42 \pm 0.03$
45.0	107.0	3.04	2.39	2.50	$0.42 \pm 0.01$	$0.39 \pm 0.03$
40.0	129.0	3.38	3.23	3.65	$0.40 \pm 0.02$	$0.43 \pm 0.03$

In all sets of parameters, the area is kept constant and equal to $A=\pi r^2+4ra =2.57$ $\hbox {m}^2$. Columns from left to right: the radius *r*, side length *a*, total length $L=2a+2r$, experimental $\gamma$ and effective $\gamma _{eff}$ control parameter, perfect $\lambda _{p}$ and standard $\lambda _{s}$ LEs.

The red open squares of Fig. 2 are the LE extracted from experimental time series for the standard BSB with a constant area $A = 2.57$ $\hbox {m}^2$. As can be seen in Fig. 2, the LEs of perfect and standard BSBs in function of $\gamma$ have the same behavior, they increase quickly reaching a maximum when $\gamma =1.0$ and decrease slowly as $\gamma \rightarrow \infty$, which makes this result the first experimental realization showing the correctness of Benettin and Strelcyn’s theoretical prediction^9^. All the quantities of interest, such as the trajectory, scattering angles, and collision times, are obtained directly from the robot footage and serve as input for the tangent method^22^, the same as that used in the numerical procedure. Each experimental value given in Fig. 2 is an average of five measurements, each calculated from a different time series recorded in a 15-minute time window. The values of the LE are given in Table 1. The characteristic lengths of the BSB are given in Fig. 1b. The experimental and numerical results are in agreement, as can be seen in Table 1.

**Figure 3:**
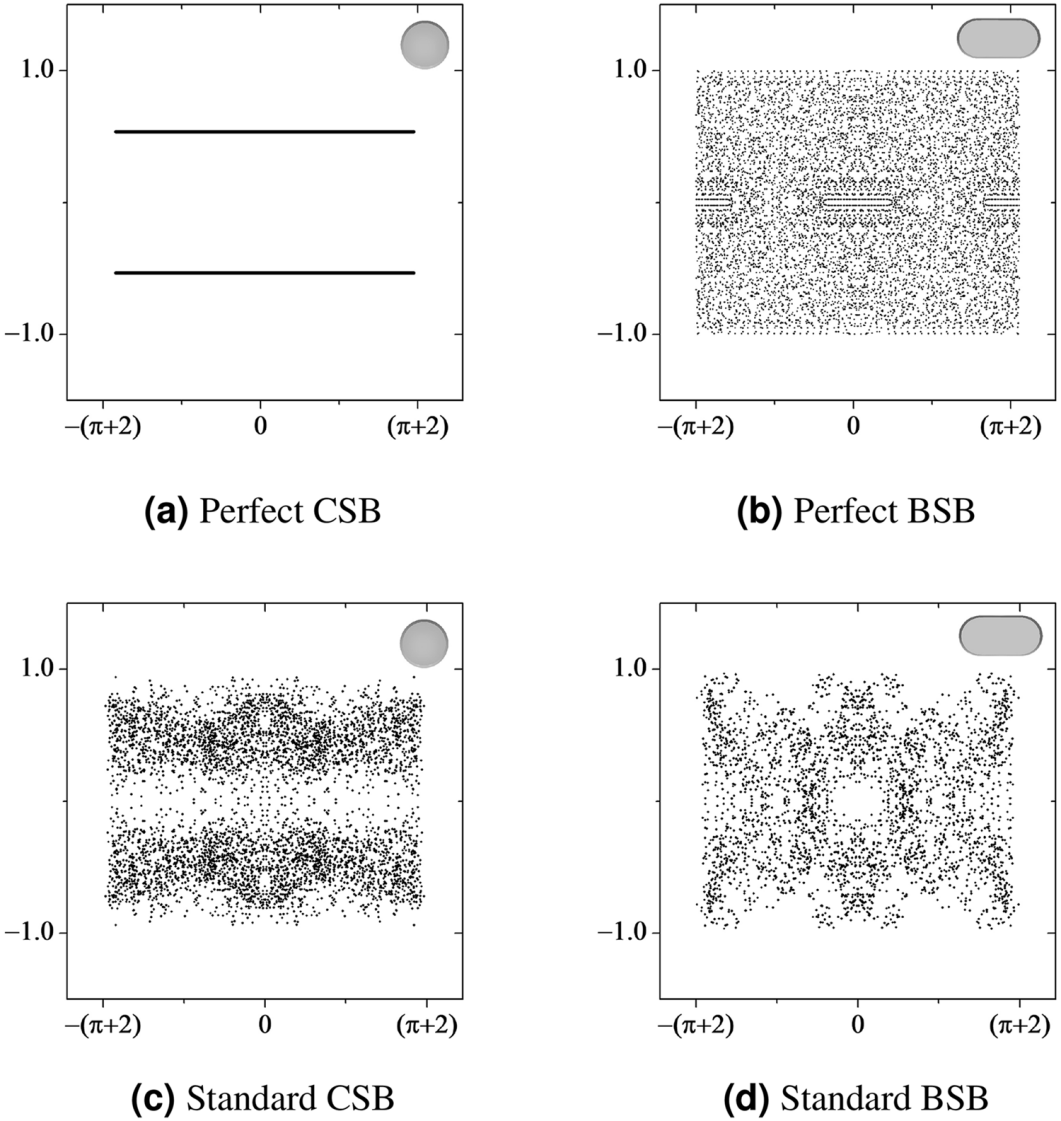
Poincaré SOS for perfect (**a**) CSB and (**b**) BSB, and standard (**c**) CSB and (**d**) BSB. We used the Birkhoff coordinates (*p*, *s*), where *p* is the arc-length at the boundary and $s={\hat{v}}\cdot {\hat{n}}=\sin {\theta _i}$ is the projection of the velocity. The domain of those variables are: $p\in [-2a-\pi r,+2a+\pi r]$ and $s\in [-1,1]$, see Fig. 1 for geometrical details. The geometrical parameters used to keep the area fixed are $r=1.51$ for CSB and $r=a=1$ for BSB.

The experimental data in Fig. 2 have outstanding characteristics that need to be pointed out. Although we know that the particle dynamics in a perfect CSB has a null LE, the robot–environment interaction in the standard CSB has a non-null LE, $\lambda = 0.03$, see Table 1. This means the standard CSB used in the experiment deviates from the geometry of a perfect CSB, in agreement with the basis of the Kolmogorov–Arnol’d-Moser theorem^5,36^. This deviation is also manifested in the maximum LE of the BSB, which happens when $\gamma =1.0$. For a perfect BSB, the LE is $\lambda =0.43$, while for the robot–environment interaction in a standard BSB, $\lambda = 0.47$. A similar deviation between perfect and standard half-mushroom cavity was also reported in optical chaos^5^. Furthermore, as will be seen, the discrepancy is more prominent in the Poincaré SOS.

Billiards, e.g., BSB, SSB, CSB, and elliptical billiards, among others, has a very rich Poincaré SOS^31–33^. It is well known that the Poincaré SOS is a smart form to reduce the dimensionality of dynamic systems^37^. The billiards has a phase space with four dimensions consisting of two spatial degrees of freedom and two conjugate momenta. Besides, the phase space of those systems has islands of stability: orbits with periodic, quasi-periodic, and chaotic behavior. However, due to conservation of the particle’s energy, the motion actually takes place on a three dimensional surface, which can be achieved by Poincaré SOS^37^. In this case it is convenient to introduce Birkhoff coordinates^34^ (*p*, *s*), where $p\in [-2a-\pi r,+2a+\pi r]$ lies on the boundary, and the projection velocity parameter $s={\hat{v}} \cdot {\hat{n}} = \sin \theta _{i}\in [-1,+1]$, where $\theta _i$ is the incident angle, as illustrated in Fig. 1d. The SOS is a set (*p*, *s*) of the collision point on the boundary^37^. As the Birkhoff coordinates preserve area and the associated Hamiltonian preserves energy, the effective dimension of the phase space for the Poincaré SOS can be reduced^37,38^. We determine the Poincaré SOS for standard CSB and BSB in order to confront the perfect CSB, which is non-chaotic, with the BSB, which is chaotic.

Additionally, the classical dynamics of a particle in a billiards have two symmetries: one related to the time reversal symmetry and the other related to the stadium billiards symmetry. The former states that the particle follows the same path if we invert the time flow, leading to an identical sequence of lengths of flights and times. On the other hand, the incident angles change sign because we define $\theta _i$ as being positive for clockwise and negative for counterclockwise. This definition of incident angle implies that to every point (*p*, *s*) in the Poincaré SOS there is another point $(p,-s)$. The latter is a consequence of the system’s geometry. BSB has a reflection symmetry in each of its two major axes. Any initial condition in the 2nd, 3rd and 4th quadrant can be mapped to the 1st quadrant, and vice versa. These two symmetries allow us to transform a single time series of the Birkhoff coordinates into effectively four time series. Therefore, the set of collision points at the billiard boundary *x*, *y* can be mapped as four time series: $(p,s)\rightarrow (p,-s)\rightarrow (-p,s)\rightarrow (-p,-s)$. In experiments using microwaves^37^ and optical cavities^5^, the number of collisions is very large, with no need to employ the symmetry of the Poincaré SOS because it emerges spontaneously from the measured data. In our case, the standard BSB has only 200 collisions and those symmetries are useful to fill the experimental (*p*, *s*) phase space.

**Figure 4:**
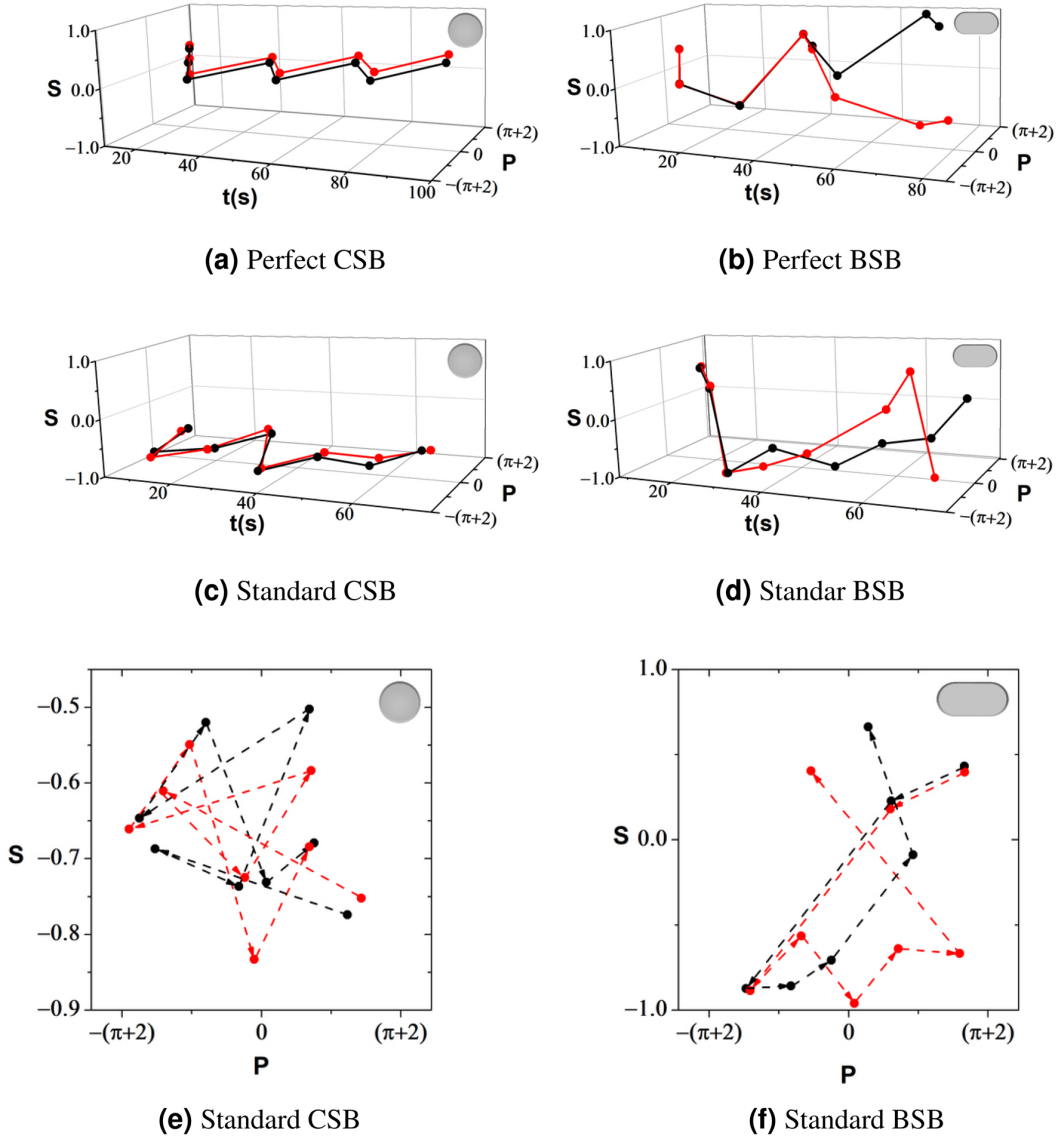
Poincaré SOS dynamics for the first 8 collisions at the wall for two close initial conditions (red and black). The plots in (**a**) and (**c**) are for a CSB, and the plots in (**b**) and (**d**) for a BSB. The red and black points in (**a**) and (**c**) are always close to each other whereas in (**b**) and (**d**) they spread apart. In (**e**) and (**f**) we show the Poincaré SOS for standard CSB and BSB with the same 8 points where we add the arrows to indicate the collision sequence

Figure 3a shows the Poincaré SOS for a perfect CSB, $\gamma =0$, which has a regular regime where the set of points (*p*, *s*) is a horizontal line, as expected^32^. This means that the angle of incidence at each collision is the same, resulting in a constant *s*. The upper horizontal line is associated with a lower horizontal line obeying the property of symmetry discussed above. Fig. 3b shows the Poincaré SOS for a perfect BSB, $\gamma =1.0$. The pattern present in the SOS corresponds to a chaotic system, with LE $\lambda = 0.43$, while for the perfect CSB it is zero. The open regions, also called islands, describe non-chaotic solutions surrounded by a chaotic sea. The islands are composed of trivial and particular solutions. The SOS for a perfect CSB and BSB are calculated from 2 and 5 slightly different initial conditions, respectively, each one with $10^{3}$ collisions.

In Fig. 3c and d are plotted the Poincaré SOS for the standard CSB ($\gamma = 0$) and BSB ($\gamma = 1$), respectively. The SOS are also calculated from 5 slightly different initial conditions, each one with 200 collisions. Fig. 3c shows an irregular regime with the set of points distributed around two horizontal straight lines, which contrasts with the regular behavior of a perfect CSB, Fig. 3a. The behavior for the standard CSB shows that the angle of incidence is different at each collision, resulting in a floating *s*, which justifies a non-null value for the LE. However, we cannot classify this as a chaotic dynamics, because these points are not distributed over the entire SOS, which means the robot will never obey an ergodic dynamics. The large differences between the Poincaré SOS of perfect and standard CSBs, Fig. 3a and c, prove that the standard billiards clearly deviates from the geometry of a perfect billiards, which makes the former more sensitive to initial conditions than the latter, in accordance with Kolmogorov–Arnol’d-Moser theorem^5,36^.

Note that Fig. 3d has a significant similarity with the SOS of a perfect BSB, Fig. 3b, which means the robot–environment interaction satisfactorily mimics the classical dynamics of particles in billiards. In addition, there is a remarkable behavior in the Poincaré SOS of the robot that deserves attention. An important difference between perfect and standard BSBs is due to the fact that the robot is an extended object, with an area of $1.75\times 10^{-3}m^2$, which is approximately $1\%$ of the total area *A*. Although this area is small, it occupies a physical region in the stadium, which has consequences. This non-null area imposes a constraint in the phase space. On the other hand, in the perfect BSB, the point-like particle is free to access the entire phase space. Naturally, the condition (*p*, *s*)=$(a+ \pi /2 r,1)$ and other symmetric points are forbidden for the standard BSB. In Fig. 3d we can observe four poorly populated regions of the SOS for the standard BSB around $p=\pm (1+\pi /2)$ and $s=\pm 1$. At the center of Fig. 3d, there is another interesting region of the Poincaré SOS. This empty central region describes a non-chaotic particular behavior, also known as a periodic solution, because the trajectories in that region are closed paths. The island of stability in the center of the SOS for the perfect and standard BSB is due to the solutions with initial positions in the rectangular region of Fig. 1b with the velocity pointing upward, i.e., ${\hat{v}}={\hat{y}}$. The trajectories are straight lines with incident angle $\theta _i=0$. The same also holds at the equator of the stadium, resulting in two islands at $p=\pm (a+\pi /2r)$ and $s=0$.

For a system in a regular regime, two realizations with infinitesimally close initial conditions stay close as time elapses. On the other hand, if the system is chaotic, a small difference between the trajectories diverges quickly. An experimental observation of those statements is naturally challenging due to the lack of control in the setup. Although, with a set of experimental data available, one can extract the distribution of the Poincaré recurrence time. The comparison between the Poincaré SOS phase space with the temporal Poincaré SOS phase space can unveil the effective properties^5^ of billiards. To probe this kind of behavior, we plot the time series of two trajectories with close initial conditions for perfect and standard CSBs through the Poincaré SOS in Fig. 4a and c, respectively. Every point is obtained only when the particle crosses the sectional plane in the phase space loop, requiring a unique per-realization number of collisions and proper times. For this reason, the plot corresponds to a time series of the Poincaré SOS. However, the two time-series stay close as time passes, guaranteeing that the CSB is non-chaotic for perfect billiards as well as in the standard billiard. In addition, Fig. 4b and d show a time series with spread points for perfect and standard BSBs, $\gamma =1$, respectively. For both cases, the two trajectories nearly coincide at the beginning but start to diverge substantially, which is a clear indication that the BSB is chaotic for perfect and standard billiards. From Fig. 4d, one can realize that after only 4 collisions the two trajectories are completely different, confirming the high sensitivity to the initial conditions of the standard billiards. Finally, Fig. 4e and f show how two trajectories propagate in the phase space for standard CSB and BSBs, respectively. In the former, the *p*-values of two trajectories remain almost constant over time, which means that they do not diverge from each other. In the latter, the *p*-values show great changes over time, which is the typical behavior of a chaotic dynamics.

**Figure 5:**
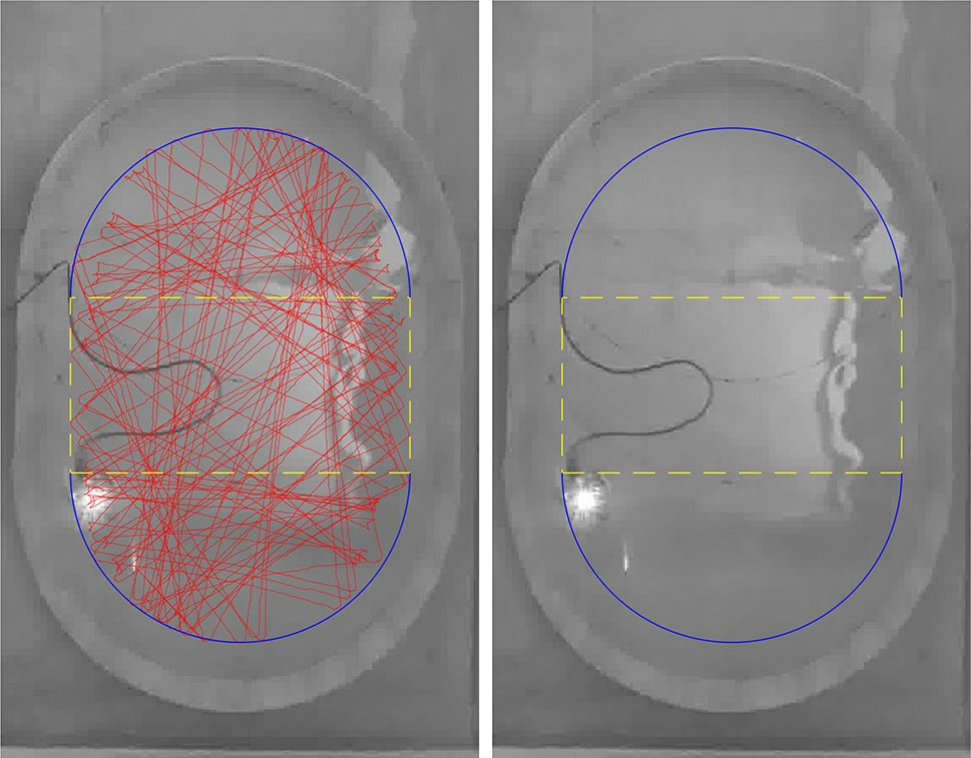
Black and white picture of the Bunimovich stadium billiards for control parameter $\gamma _{\mathrm{eff}}=0.52$, Table 1. In red we show the robot trajectory over 15 mins, recorded at 30 fps. In blue we show the effective semi-circular boundaries and in yellow the central rectangle with side length 2*a*.

## Experimental setup

We performed measurements in six different standard BSBs by capturing footage of the robot’s movement for fifteen minutes. For each setup, we carried out five different video recordings with similar initial conditions. The length of the stadium ranges from 1.80 m, for the CSB, to 3.38 m for the most elongated BSB. The geometric parameters used in the experiments are described in Table 1 and illustrated in Fig. 1b. We used MDF industrial wooden sheets 25 cm high to make the walls of the stadiums.

The robot is an Arduino controlling board powered by a USB cable and integrated with two step-motor engines and three infrared (IR) sensors, Fig. 1c. The dimensions of the robot are: 14 cm long, 12.5 cm wide, and 16 cm high. The video recordings were made by a full HD resolution camera at @30 fps, positioned 2.80 m above the center of the stadium. We collected approximately 27, 000 images of the robot per recording. That is the number of points of the robot’s trajectory in the (*x*, *y*)-plane. For the acquisition of the trajectory data, we employed a Matlab customized code using a tracking algorithm. Figure 5 shows a black and white picture of experiment for the standard BSB with a robot’s trajectory in the (*x*, *y*)-plane (red line) obtained by the tracking algorithm. Furthermore, we can see the stadium with its real dimension (wooden wall) and effective dimension (blue and yellow lines). The code is simple but with high efficiency because we do not have occlusions or several targets objects in the scenario. In this way, no frames obtained in experiment footage are wasted, and the robot’s position time series has no gaps. The average velocity of the robot was 14.2 cm/s, which means 2 frames/cm, or more specifically, a spatial error of $\pm 5$ mm.

There is a gap between the IR sensors and the central LED spot, causing the tracking algorithm to detect only an effective area of the stadium. Thus, to consider the actual dimensions of the stadium, certain adjustments in the collected trajectory data are necessary. Figure 5 shows a picture of the stadium with its actual and effective dimensions. To take this offset length of approximately 5 cm into account, we directly measured the ratio between the effective radius *r* and side length *a* from the robot’s trajectory. This procedure is only correct if the number of collisions between the robot and the boundaries is large enough. The number of collisions is proportional to the recording time. In our procedure, this number is always larger than one hundred. We also observed that the effective $\gamma _{\mathrm{eff}}$ is always around $5\%$ larger than the constructed $\gamma$, see Table 1.

As shown in Fig. 5, the effective area must be the smallest BSB enclosing the experimental data. The calculation of the effective $\gamma _{\mathrm{eff}}$ depends on the choice of three free parameters. Naturally, we have the effective radius *r* and the side length *a* to be determined. An additional parameter, a rotation angle between the stadium and the camera alignment appears in the experiment. To find these three parameters, we used the minimum bounding box algorithms^39^. These can connect the lengths of the rectangle with the radius and side length of the stadium, giving rise to $\gamma _{\mathrm{eff}}$, see Table 1.

## Theoretical approach

In this section, we briefly introduce the tangent space method used to calculate the LE shown in Fig. 2. The LE measures the degree of divergence between nearby orbits in the state space^40^. Let us consider ${\mathbf {x}}\equiv (x_1, x_2, \dots , x_\nu )$ as the coordinates in a $\nu$-dimensional phase space. The system evolves according to an autonomous first-order differential equation, given by

1 $$\begin{aligned} \frac{d{\mathbf {x}}(t)}{dt} = {\mathbf {F}}({\mathbf {x}}(t)), \end{aligned}$$

where ${\mathbf {F}}({\mathbf {x}})$ is the velocity field corresponding to the solution flow of Eq. (1). Thus, expansions/contractions of the trajectories in a neighborhood of ${\mathbf {x}}(t)$ are estimated through two different solutions, ${\mathbf {x}}^{(1)}(t)$ and ${\mathbf {x}}^{(2)}(t)$, of Eq. (1). From ${\mathbf {x}}^{(1)}(t)$ and ${\mathbf {x}}^{(2)}(t)$, we determine the evolution of the difference vector ${\mathbf {w}}\equiv {\mathbf {x}}^{(1)}(t) - {\mathbf {x}}^{(2)}(t) \equiv (\delta x_1, \delta x_2, \dots , \delta x_\nu )$. In an infinitesimal regime, the equations for this deviation vector are linear, given by the first variation equation

2 $$\begin{aligned} \frac{d{\mathbf {w}}(t)}{dt} = {\mathbf {J}}({\mathbf {x}}(t)){\mathbf {w}}(t), \end{aligned}$$

with ${\mathbf {J}}$ being the $\nu \times \nu$ Jacobian matrix with respect to the vector field ${\mathbf {F}}({\mathbf {x}}(t))$. Since the elements of ${\mathbf {J}}$, provided by (1), are continuous bounded function of *t* for $t\rightarrow \infty$, the solutions of the linearized Eq. (2) grow at most as an exponential function. For any initial perturbation ${\mathbf {w}}(0)$, the growth rate

3 $$\begin{aligned} \lambda = {\displaystyle \lim _{t\rightarrow \infty } \ln \frac{|{\mathbf {w}}(t)|}{|{\mathbf {w}}(0)|}} \end{aligned}$$

is defined as the LE. In terms of the definition (3), one can characterize each eigendirection of the system. The set of all exponents obtained, which is called the Lyapunov spectrum, is usually ordered as $\lambda _1 \ge \lambda _2 \ge \dots \ge \lambda _\nu$^21^. Denoted by $\lambda _1$, the largest LE indicates whether ${\mathbf {x}}(t)$ stabilizes, when the limit (3) exists. The presence of divergent (convergent) trajectories and therefore a chaotic (regular) regime is indicated by positive (negative) exponents. This theoretical framework constitutes an introduction to a powerful tool for detecting and quantifying chaos. Analytically using the differential geometry approach, there are investigations where the LE is determined from the properties of the curvature of the Riemannian manifold^41,42^. From the long-time average (3), and arguments of ergodicity, it can also be obtained computationally, via the numerical integration of the deviations ${\mathbf {w}}(t)$ evaluated along a trajectory ${\mathbf {x}}(t)$^21,43^.

In the context of the experimental data of discrete measurements, techniques of phase space reconstruction with delay coordinates make it possible to access values of the LE comparable to those of the original attractor^23^. The linearized version of the system is not defined for this experimental approach, in which the deviations are calculated by direct subtraction between close orbits in the reconstructed phase space. Thus, one assumes the deviations to be small enough, preserving the magnitude and direction properties of the measurements generated from the tangent space.

In the present work, our experimental setup was constructed to provide all the measurements required by the Jacobian (2). As a consequence, the LE is obtained from the tangent space, without the need for phase space reconstruction and also without approximations to define the deviation vector. For the robot, the deviation vectors evolve according to two different cases of the linearized equations. One of them is when there are no collisions, indicated by the Jacobian:

4 $$\begin{aligned} {\mathbf {J}}_0= \begin{bmatrix} &{}1 &{} &{}\tau &{}\\ &{}0 &{} &{}1&{} \end{bmatrix}, \end{aligned}$$

with $\tau$ representing the time between two collisions. The other one sets the behavior of the deviations at the collisions, in which we have

5 $$\begin{aligned} {\mathbf {J}}_c= -\begin{bmatrix} &{}1 &{} &{}0 &{}\\ &{}{\displaystyle \frac{2\kappa }{\cos {\varphi }}} &{} &{}1&{} \end{bmatrix}, \end{aligned}$$

where $\kappa$ is the curvature of the boundary point at which the collision occurs and $\varphi$ is the angle of incidence of the particle with relation to the boundary. For a stadium, the curvature assumes two different values. We have $\kappa = 0$ when the collision is located in the rectangular region and otherwise $\kappa = 1/r$, the curvature of the circular part. As a limitation, our method to calculate LE from experimental time series requires linearized equations related to dynamics that emulate the experimental setup.

## Conclusions

In this paper, we mimicked the dynamics of a particle in perfect Bunimovich stadium billiards (BSB) by a robot–environment interaction in a standard BSB. We carried out six different experiments with standard BSBs with fixed area and determined the values of the Lyapunov exponent (LE) using the tangent space method. The LEs from our experimental data were confronted with well-established theoretical results of Benettin and Strelcyn^9^, with a satisfactory agreement.

We also determined the Poincaré SOS of the billiards, which indicates a deviation between the geometry of perfect and standard BSBs. Finally, to show the sensitivity to the initial conditions and as an experimental control, we constructed the Poincaré SOS as a function of time, Fig. 4.

We stress that our focus here is on presenting an experimental setup to mimic a highly idealized model through programming the robot’s responses, as close as possible, to the validity conditions of the model. However, the robot-environment interaction occurs in much broader scenarios such as gesture communication between human and robot^44^, speech emotion recognition^45^, finding pattern hidden in data-sets^46,47^, and gender identification from speech^48^ just to cite a few.

This work paves the way to proposals of idealized models to these more complex situations. Idealized models help us deepen our understanding of complex realities, and they shed light on mechanisms hidden underlining subtle behaviors.

In conclusion, our results indicate that robot–environment interaction is a great platform to experimentally study chaotic dynamics, which makes it possible to test other theoretical predictions.
